# Radiotherapy and DNA damage response inhibitors modestly sensitize HNSCC to NK cell killing, with ATM inhibition more effective than ATR inhibition

**DOI:** 10.1007/s00066-026-02522-3

**Published:** 2026-04-17

**Authors:** Leonie Steinsdörfer, Lilli Zülch, Anna Schäfer, Benjamin Frey, Stefanie Corradini, Rainer Fietkau, Udo S. Gaipl, Tina Jost

**Affiliations:** 1 Translational Radiobiology, Department of Radiation Oncology, Uniklinikum Erlangen, Friedrich-Alexander-Universität Erlangen-Nürnberg, Erlangen, Germany; 2 Comprehensive Cancer Center Erlangen-EMN, Erlangen, Germany; 3 FAU Profile Center Immunomedicine (FAU I-MED), Friedrich-Alexander-Universität (FAU), Schlossplatz 1, 91054 Erlangen, Germany; 4 Department of Radiation Oncology, Uniklinikum Erlangen, Friedrich-Alexander-Universität Erlangen-Nürnberg, Erlangen, Germany

**Keywords:** Combination therapy, HPV, Co-culture, Immunomodulation, Innate immune system

## Abstract

**Purpose:**

Human papillomavirus (HPV)-negative head and neck squamous cell carcinomas (HNSCC) remain challenging due to their reduced radiosensitivity. Targeting the DNA damage response (DDR) with kinase inhibitors may enhance tumor susceptibility to radiotherapy (RT). While RT is known to alter the immune phenotype of HNSCC cells, its impact on innate immune responses, particularly on natural killer (NK) cells, remains unclear. We therefore investigated how RT combined with ATM or ATR inhibition—key DDR kinases—affects NK cell-mediated killing and activation.

**Methods:**

The tumor cell lines HSC4 and Cal33 (both HPV-negative) and UM-SCC-47 and UD-SCC‑2 (both HPV-positive) were treated with the DDR inhibitors VE-822 (ATRi) and AZD0156 (ATMi) and hypofractionated RT (2 × 5 Gy). After co-cultivation with primary human NK cells, tumor cell death was measured using flow cytometry after 24 h. Further, NK cell activation markers and the supernatant of tumor cell–NK cell co-cultures were measured and the effect of immune checkpoint inhibitors (durvalumab, monalizumab) was additionally investigated.

**Results:**

Particularly the HPV-negative HSC4 cells pretreated with either ATMi or RT + ATMi showed significantly increased killing by NK cells. Reduced secretion of granulysin by NK cells was mainly found in the co-cultures of HPV-negative HNSCC treated with ATRi. Treatment with the immune checkpoint inhibitor durvalumab (anti-PD-L1) did not result in significant augmentation of NK cell-induced tumor cell killing in this setting. Conversely, treatment with monalizumab (anti-NKG2A) resulted in a modest increase.

**Conclusion:**

Natural killer cells showed only a limited contribution to the killing of HNSCC cells pretreated with RT or RT + DDRi. However, a subset of patients with head and neck tumors—such as those represented by the HSC4 model—might still benefit from combining RT with ATMi rather than ATRi to enhance NK cell-mediated tumor killing.

**Supplementary Information:**

The online version of this article (https://doi.org/10.1007/s00066-026-02522-3) contains supplementary material, which is available to authorized users.

## Introduction

Head and neck squamous cell carcinoma (HNSCC), located in the oral cavity, pharynx, and larynx, is the most common type of cancer occurring in the head and neck region, with both incidence rates and mortality rising globally over the past decade [1, 2]. Conventional treatment of head and neck cancer typically involves a multimodal approach, including surgery, radiotherapy, and systemic treatment such as chemo- and immunotherapy. These treatments require intensive supportive care before, during, and after treatment and are frequently associated with substantial side effects. Despite this, relapse rates remain high, underscoring the need for novel therapeutic strategies to improve patient survival and clinical outcome [3, 4].

Head and neck squamous cell carcinoma comprises a highly heterogeneous group of malignancies that can be classified into distinct subgroups based on their etiology. Tobacco and alcohol consumption, either independently or in combination, are well-established major risk factors HNSCC development [5]. However, tumors in the oropharynx are increasingly linked to human papillomavirus (HPV) infections, mostly the oncogenic subtypes HPV-16 and, to a lesser extent, HPV-18 [1]. These HPV-negative and HPV-positive tumors show major molecular differences that result in clinical differences, and HPV-positive HNSCC is associated with a better prognosis overall [6]. The reason for this is often increased radiosensitivity of HPV-positive tumors due to downregulation of the DNA double-strand break (DSB) repair system, leading to higher cell death through DNA damage induced by radiotherapy (RT) [7]. Thus, possibilities to enhance the susceptibility to irradiation, especially of HPV-negative tumors, are sought. One promising treatment option consists of using small molecule kinase inhibitors (smKIs), blocking certain key molecules of the DNA damage response (DDR) [8, 9]. Two important regulators of the DDR pathway are the ataxia-telangiectasia mutated (ATM) and ATM/Rad3-related (ATR) proteins, both of which belong to the phosphoinositide 3‑kinase (PI3K) family and can be targeted by the drugs AZD0156 (ATM inhibitor) and VE-822 (ATR-inhibitor), respectively [10]. Additional inhibition of suppressive immune checkpoint molecules (ICM) can further strengthen antitumor responses by fostering dendritic cell-dependent T cell activation against the tumor [11].

Completed phase I studies on AZD0156 in advanced solid tumors have already shown radiosensitizing effects, for example in melanoma cells, and enhanced effects of the poly(ADP-ribose) polymerase (PARP) inhibitor olaparib across cancer cell lines from different entities [12–14]. VE-822 is being investigated in phase I and II clinical trials and seems to lead to a better treatment response in small cell lung cancer, pancreatic ductal adenocarcinoma, and other advanced solid tumors [15–19]. Furthermore, ATR inhibition (ATRi) in combination with RT achieves both tumor control and immune activation [20]. Preclinically, we have already shown increased tumor cell death and senescence induction when combining ATM and ATR inhibition with ionizing radiation [9, 21]. We also investigated alterations in the immune phenotype of HNSCC cells induced by ATM and ATR inhibition in combination with hypofractionated radiotherapy and revealed increased surface expression levels of immunostimulatory ICMs such as inducible costimulatory ligand (ICOS-L) and CD137‑L, the ligand for CD137 that is expressed on activated T and NK cells, on HNSCC cells, particularly by combining RT with ATRi [22]. These surface ICM contribute to regulation of the antitumor response by being able to recruit and activate certain kinds of immune cells, including T cells [23]. However, there is still limited knowledge regarding how this impacts innate immune cells such as natural killer cells (NK cells), even though both ICOS‑L and CD137‑L are linked to the activation and functionality of NK cells [24, 25].

Natural killer cells play a pivotal role in immune surveillance and the elimination of cancer cells, as they possess the ability to recognize and directly target transformed or infected cells without the need for prior sensitization and independently of the major histocompatibility complex (MHC) [26]. They are able to infiltrate tumors, release cytokines, and kill target cells on their own and therefore have high potential for new therapeutic strategies [27]. We have already demonstrated alterations in the expression of activity-associated receptors on NK cells such as NKG2D, NKp46, NKp44, and NKp30 after co-cultivation with pretreated HNSCC tumor cells, particularly of those that were treated with RT and inhibition of ATM (ATMi) [28]. Nevertheless, it remains to be investigated whether this also leads to alterations in the functionality and killing ability of NK cells. Additionally, studying NK cells offers valuable insights into potential immunotherapeutic strategies, such as the use of monoclonal antibodies against different inhibitory checkpoints. Expressed not only by T cells but also by NK cells, André et al. showed that NKG2A may represent a promising immune checkpoint in cancer treatment [29]. In animal models of HNSCC, compared with ATRi plus RT treatment alone, adding concurrent NKG2A and PD-L1 blockade in the adjuvant, postradiotherapy setting elicits a strong antitumor response characterized by increased infiltration and activation of cytotoxic T cells within the tumor microenvironment [30].

To investigate the importance of NK cells in the anti-HNSCC response to pretreated HNSCC tumor cells, we analyzed how the two key inhibitors of the DDR (ATMi and ATRi) interact with hypofractionated RT (2 × 5 Gy) with regard to NK cell activation and functionality after co-culture with the respectively treated HNSCC tumor cells. We hypothesized that co-culturing these HNSCC tumor cells with primary human NK cells differentially affects the ability of these innate immune cells to kill the HNSCC cells and that this can be further modulated by immune checkpoint inhibitors targeting the PD-L1/PD axis or NKG2A on the NK cell surface.

## Materials and methods

### Cell lines and cell culture

Four human HNSCC cell lines were investigated: Cal33 and HSC4 as HPV-negative and UM-SCC-47 and UD-SCC‑2 as HPV-positive lines. The cell lines were cultured in Dulbecco’s modified Eagle’s medium (DMEM; PAN-Biotech GmbH, Aidenbach, Germany) supplemented with 10% fetal bovine serum (FBS superior; Sigma-Aldrich, Darmstadt, Germany) and 1% penicillin-streptomycin (PenStrep; Gibco, Waltham, MA, USA). They were grown in Cellstar® cell culture flasks (Greiner Bio-One International GmbH, Kremsmünster, Austria) and passaged twice per week using 0.5% trypsin solution (Gibco, Waltham, MA, USA). Primary NK cells isolated from healthy human donors were cultured in RPMI-1640 medium (Sigma-Aldrich, Darmstadt, Germany) supplemented with 10% FBS and 1% PenStrep as “NK cell medium” and stored in 48-well cell culture plates (Greiner Bio-One International GmbH, Kremsmünster, Austria). All cells were cultivated at 37 °C in humidified air with 5% CO_2_.

### Treatments and irradiation scheme

The tumor cells were seeded into two 48-well plates (Greiner Bio-One International GmbH) at a number adjusted to the different cell lines to reach 70–80% confluency and were stained after 24 h with carboxyfluoreszeindiacetat-*N*-succinimidylester (CFSE; Sigma-Aldrich). Thereafter, they were treated with either 1 µM of the ATM inhibitor AZD0156 (Selleckchem, Houston, TX, USA) or 0.1 µM of the ATR inhibitor VE-822 (Selleckchem), which were both dissolved in dimethyl sulfoxide (DMSO; Roth, Karlsruhe, Germany). The control cells were treated with DMSO only. Irradiation was performed by using an Isovolt Titan X‑ray tube (GE, Ahrensburg, Germany) following a hypofractionated scheme consisting of two doses of 5 Gy each (120 keV). The first dose was applied 3 h after treatment with the DDRi, the second dose 24 h after the first one.

### Isolation of NK cells from peripheral blood mononuclear cells

Primary human NK cells were isolated from healthy donors, who varied in the different experiments. First, peripheral blood mononuclear cells (PBMCs) were collected using density gradient centrifugation in SepMate^TM^-50 PBMC Isolation Tubes (Stemcell, Vancouver, Canada). The PBMCs were centrifuged and resuspended in Dulbecco’s phosphate-buffered saline (DPBS; Gibco) with 2% FBS (Sigma-Aldrich) twice, and the amount of PBMCs was calculated using a Neubauer counting chamber (Paul Marienfeld GmbH & Co. KG, Lauda-Königshofen, Germany) in a dilution of 1:10 with trypan blue (Sigma-Aldrich). Thereafter, 1 × 10^7^ cells were resuspended in 40 µL MACS buffer consisting of 500 mL PBS (PBS tablets pH 7.4, 500 ml/tablet; Medicago AB, Uppsala, Sweden), 10 mL EDTA disodium salt dihydrate (Roth), and 25 mL MACS® BSA stock solution (Miltenyi Biotec, Bergisch Gladbach, Germany). Natural killer cells were isolated from the PBMCs using the NK Cell Isolation Kit from Miltenyi Biotec. For this, the PBMCs were first incubated in 10 µL per 10^7^ cells of NK isolation biotin-conjugated antibodies (Miltenyi Biotec) against different surface markers not expressed on NK cells and stored for 10 min at 4 °C. Then 30 µL per 10^7^ cells of MACS buffer and 20 µL per 10^7^ cells of the magnetic NK isolation beads (Miltenyi Biotec) were added and the cells were stored again at 4 °C for 15 min. The cell–beads cocktail was pipetted onto a MACS® column (Miltenyi Biotec) which was placed in a magnet (Miltenyi Biotec) and had been prewashed with 500 µL of MACS buffer, causing magnetically labelled cells to stick to the magnet. The unlabeled NK cells were collected in the flowthrough via negative selection. They were counted using the Neubauer counting chamber and a dilution of 1:4 with trypan blue, centrifuged, and resuspended in NK cell medium to a concentration of 2–3 × 10^6^ cells/mL. After stimulation with 100 U/mL interleukin (IL)-2 (Bio-Techne, Minneapolis, MN, USA) and 25 ng/mL IL-15 (Thermo Fisher Scientific), the cells were incubated for 24 h. At the outset of the study, NK cell viability and activation status were systematically assessed immediately after isolation and at 24, 48, and 72 h following stimulation with IL‑2 and IL-15. Activation marker expression increased up to 48 h of stimulation, whereas viability progressively declined thereafter, with marked cell death occurring beyond 48 h (data not shown).

### Co-culturing of pretreated HNSCC tumor and NK cells

**Fig. 1:**
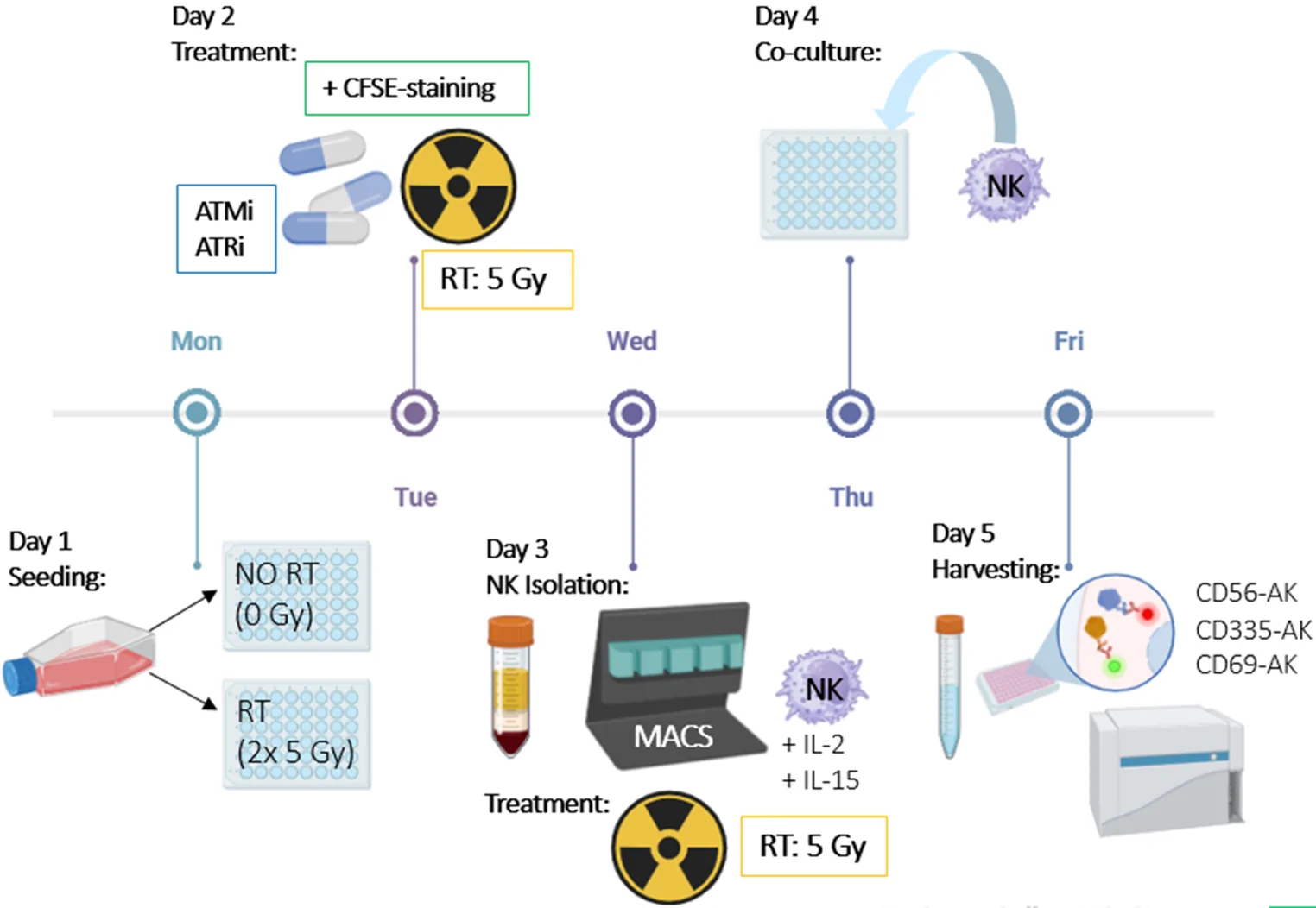
Experimental design of treatment and co-cultivation of pretreated HNSCC and NK cells. HPV-negative (HSC4, Cal33) and HPV-positive (UM-SCC-47, UD-SCC-2) HNSCC cells were seeded on day 1, stained with CFSE, and treated with 1 µM AZD0156 or 0.1 µM VE-822 on day 2. Irradiation with a single dose per fraction of 5 Gy was performed 3 h after treatment with the inhibitors on day 2 and repeated 24 h later on day 3, where primary human NK cells were also isolated from healthy donors. Pretreated HNSCC tumor cells and NK cells were co-cultivated in a 1:1 ratio on day 4. Cells were harvested and analyzed 24 h after co-culturing on day 5. Image created with BioRender. *ATMi* Ataxia Telangiectasia Mutated Protein Inhibitor, *ATRi* taxia telangiectasia and Rad3-related Protein Inhibitor, *CFSE* Carboxyfluorescein succinimidyl ester, *Gy* Gray, *HNSCC* Head and neck squamous cell carcinoma, *NK* natural killer cells, *RT* radiotherapy

Prior to co-culture, the DMEM medium of the tumor cells was replaced to prevent NK cells from being exposed to the DDRi (ATMi or ATRi). A sample of tumor cells from each treatment group was harvested to check the number of cells still viable after treatment using the CasyCount (OMNI Life Science GmbH & Co KG, Bremen, Germany). The NK cells were harvested and resuspended in NK cell medium to a concentration of 1000 cells per µL. The calculated number of NK cells was pipetted onto the tumor cells in a one-to-one ratio. A 1:1 NK-to-tumor-cell ratio was used to provide standardized and reproducible conditions, enhancing assay sensitivity for detecting subtle treatment-induced changes in tumor cell susceptibility while accounting for the high variability of NK cell infiltration in vivo. One well of each treatment group was left without NK cells for comparison. The co-cultures were incubated for another 24 h and harvested for measurement the next day. The workflow is depicted in Fig. 1.

For subsequent experiments, the same workflow was performed, but on day 4, immune checkpoint inhibitors were additionally added: the PD-L1 inhibitor durvalumab (AstraZeneca GmbH, Cambridge, UK) was used in a concentration of 6.02 mg/mL and the NKG2A inhibitor monalizumab (Selleckchem, Houston, TX, USA) at 7.72 mg/mL.

### Flow cytometry analysis of NK cell activation markers and tumor cell killing

**Fig. 2:**
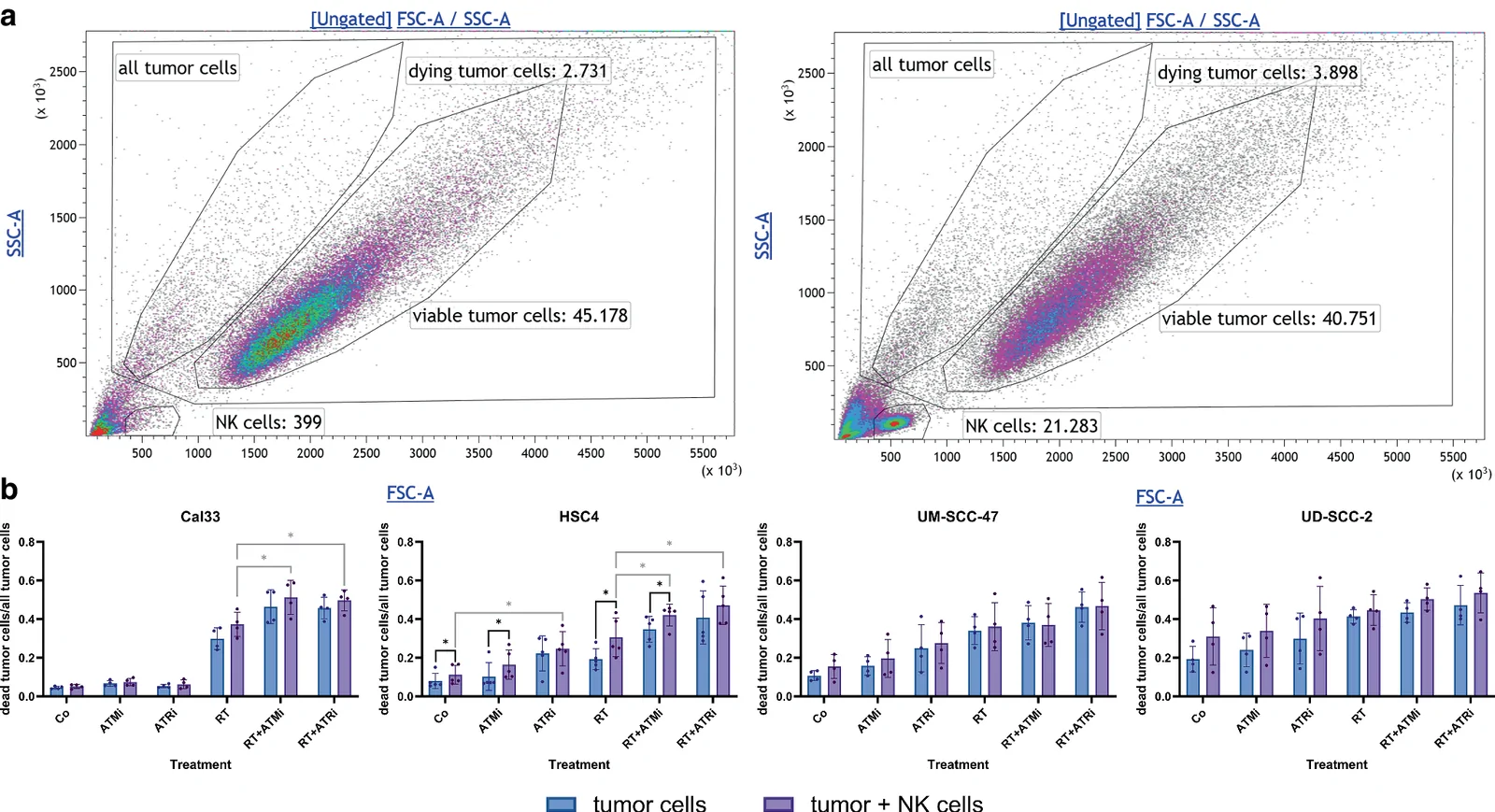
Cell death rates of pretreated HNSCC after co-cultivation with primary human NK cells for 24 h. **a** Gating strategy for defining the respective cell populations: NK cells (CD56+), viable tumor cells (CFSE+), and dying tumor cells (CFSE+) were differentiated by cell size and surface granulation using the forward (*FSC*) and side scatter (*SSC*). In the first dot plot, the gating is exemplarily shown for the tumor cell control group without NK cells in the HSC4 cell line, in the second one for the corresponding tumor cell–NK cell co-culture. The numbers represent the cell count of each gate. **b** Tumor cell killing in the four investigated HNSCC cell lines for the different treatment conditions, each with and without NK cells. The HNSCC cell lines Cal33, HSC4, UM-SCC-47, and UD-SCC‑2 were pretreated with either 1 µM of the ATM inhibitor AZD0156, 0.1 µM of the ATR inhibitor VE-822, RT of 2 × 5 Gy, or a combination. DMSO-exposed cells cultures were used as control (*Co*). Cell death rates with and without NK cells were measured in each treatment group using multicolor flow cytometry as described in Fig. 2a. Each value shows mean ± SD (Cal33, *n* = 4; HSC4, *n* = 5; UM-SCC-47, *n* = 4; UD-SCC‑2, *n* = 4; all replicates were performed with NK cells from individual donors). Significance was determined by one-tailed Mann–Whitney U test; *p ≤ 0.050. *ATM* ataxia telangiectasia mutated Protein, *ATR* ataxia telangiectasia and rad3-related Protein, *CFSE* Carboxyfluorescein Succinimidyl Ester, *DMSO* Dimethyl Sulfoxide, *HNSCC* Head and Neck Squamous Cell Carcinoma, *NK* natural killer cell

The analysis was performed using multicolor flow cytometry and the associated Kaluza 2.3 Software® (Beckman Coulter, Brea, CA, USA). The samples were harvested, centrifuged, and the supernatants were collected for further analyses, as described below. After centrifugation the cells were stained with a mastermix of the following antibodies: anti-CD56 (mouse IgG2b CD56 antibody, APC-R700; BD Biosciences, Franklin Lakes, NJ, USA), anti-CD335 (anti-human CD335 antibody, PC5; Beckman Coulter), and anti-CD69 (mouse IgG1 CD69 antibody; PE-Vio 770, Miltenyi Biotec) and stored for 30 min at 4 °C. After another centrifugation, each sample was resuspended in 100 µL FACS buffer consisting of PBS (Medicago AB), 2% FBS (Sigma-Aldrich), and 2% EDTA disodium salt dihydrate (Roth). Surface marker expression was measured with the Dx-Flex Cytometer (Beckman Coulter). Forward scatter (FSC) and side scatter (SSC) were used to differentiate the different cell populations, including the dead tumor cells, as shown in Fig. 2. For all HNSCC cell lines, four to five independent experiments were performed (Cal33, *n* = 4; HSC4, *n* = 5; UM-SCC-47, *n* = 4; UD-SCC‑2, *n* = 4).

### Analysis of NK cell-related molecules in the supernatants

The collected supernatants were frozen at −20 °C until all experiments were finished. Then the LEGENDplex™ Human CD8/NK Panel from BioLegend (San Diego, CA, USA) was performed. The bead-based multiplex assay uses fluorescence-encoded beads and allows quantification of IL‑2, IL‑4, IL‑6, IL-10, IL-17A, interferon (IFN)-γ, tumor necrosis factor (TNF)-α, Fas, Fas ligand, granzyme A, granzyme B, perforin, and granulysin. Into each well of a 96-well plate, 25 µL of assay buffer (BioLegend) and 25 µL of either standard or sample was pipetted; 25 µL of mixed beads (BioLegend) were added and the plate was shaken at 800 rpm on a plate shaker for 2 h at room temperature. The plate was centrifuged, washed with 1X Wash Buffer (BioLegend), and 25 µL of detection antibodies (BioLegend) were added. After shaking at 800 rpm on a plate shaker for 1 h, 25 µL of streptavidin-phycoerythrin (SA-PE; BioLegend) was added, followed by another 30 min on the plate shaker. After centrifugation, washing, and resuspension in 1X Wash Buffer, the samples were analyzed on the Dx-Flex Cytometer (Beckman Coulter).

### Statistical analysis

Statistical analyses were performed using Graph Pad Prism (version number 9.5.1; GraphPad software, Inc., San Diego, CA, USA). All data are presented as mean ± SD. Significance was determined by one-tailed Mann–Whitney U test and set at *p* ≤ 0.050.

### Ethics statement

This assay involves blood withdrawals and cultivation of human primary cells. All results presented in this manuscript are covered by the ethical approval of the IMMO-NHD trial, and written informed consent was obtained from all donors. Approval was granted by the institutional review board of Friedrich-Alexander-Universität Erlangen-Nürnberg on November 9, 2022 (application number 21-415-B).

## Results

### Pretreating HSC4 tumor cells with ATMi or RT + ATMi significantly increases NK cell-induced tumor cell killing

Meidenbauer et al. showed that the treatment of HNSCC with ATMi or ATRi alone and in combination with RT modulates the immune phenotype of HNSCC cancer cells [22]. However, since both immune-stimulatory and immune-suppressive ICM were modulated, it was logical to consecutively examine how this impacts tumor-targeting immune cells such as NK cells. We therefore first established an NK cell-killing assay to investigate both expression of activation markers on NK cells and NK cell-dependent tumor cell killing. After co-culturing pretreated tumor cells and NK cells for 24 h, tumor cell death rates were measured via flow-cytometry, differentiating the three cell populations—NK cells, viable tumor cells, and dying tumor cells—by their difference in cell size and surface granulation, as shown in Fig. 2a.

We particularly investigated the impact of the DDRi AZD0156 (ATMi) and VE-822 (ATRi) on the cytotoxicity of NK cells by measuring cell death rates of the four different HNSCC cell lines after pretreating them with either ATMi or ATRi, hypofractionated RT of 2 × 5 Gy, or a combination (Fig. 2b). To demarcate NK cell-induced tumor cell killing from tumor cell death due to treatment, an equally treated control without NK cells was performed for each treatment group.

For the two HPV-positive cell lines UM-SCC-47 and UD-SCC‑2, no significant effect of the NK cell cytotoxicity was found in either treatment group. Regarding the HPV-negative cell lines, Cal33 showed a significant increase in tumor cell death induced by the DDRi in combination with RT compared to RT alone, but also no significant effect on NK cell-induced tumor cell killing. In HSC4, however, a significant increase in NK cell-induced tumor cell killing of tumor cells treated with ATMi, RT, or RT + ATMi was observed. This was not seen for pretreatment of the HSC4 cells with ATRi or RT + ATRi. While our previous work has already revealed that the response to treatment of HNSCC with ATMi or ATRi is also cell line dependent [22], again, the NK cell-induced tumor cell killing was significant in only one HPV-negative tumor cell line.

### Reduced release of granzyme b and granulysin particularly after pretreatment of HSC4 tumor cells with ATRi

**Fig. 3:**
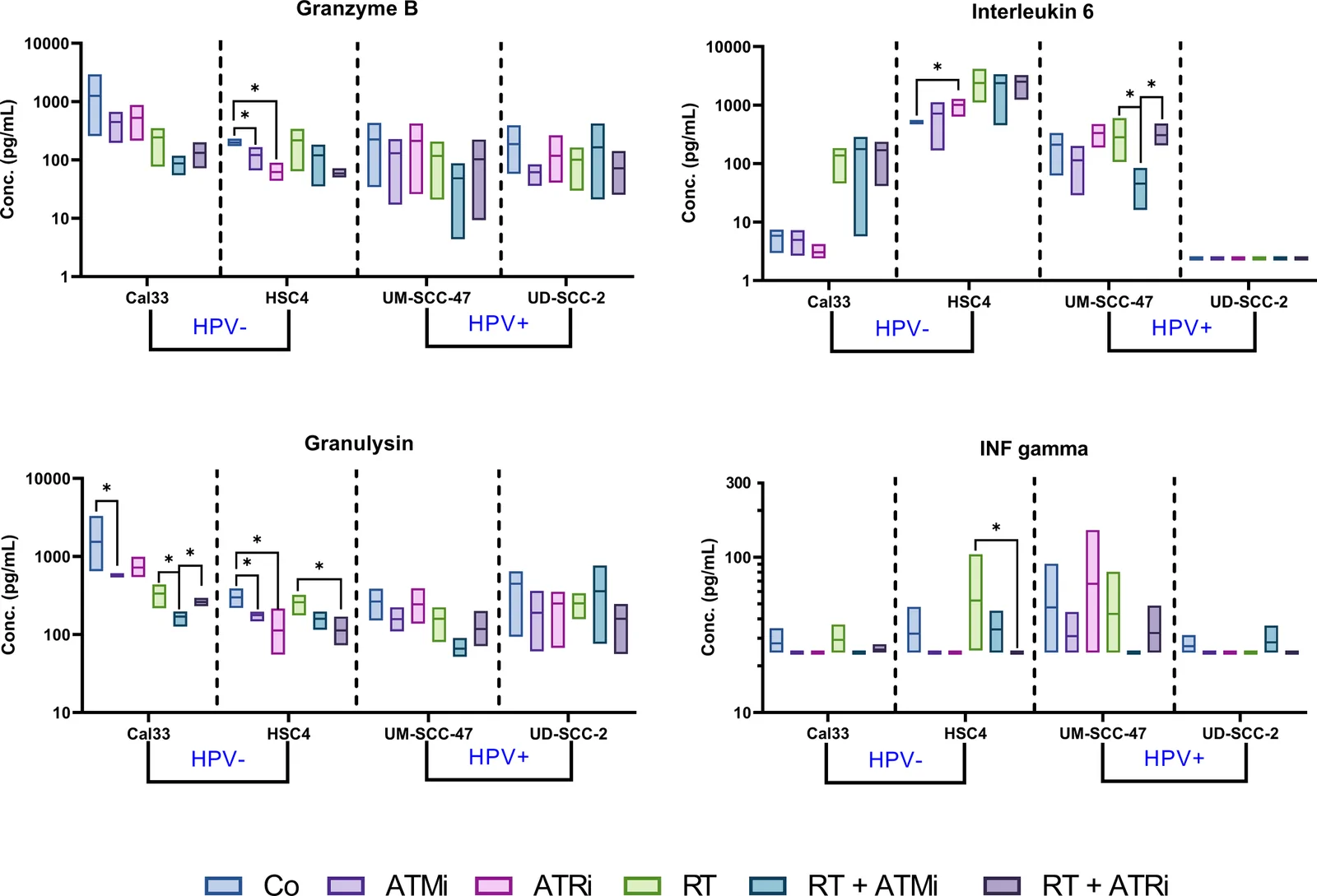
Secretion of NK cell-related molecules after co-cultivation with pretreated HNSCC for 24 h. The HNSCC cell lines Cal33, HSC4, UM-SCC-47, and UD-SCC‑2 were pretreated with either 1 µM of the ATM inhibitor AZD0156, 0.1 µM of the ATR inhibitor VE-822, RT of 2 × 5 Gy, or a combination. DMSO-exposed cell cultures served as control (*Co*). Secretion of granzyme B, IL‑6, granulysin, and INF‑γ was analyzed using a bead-based multiplex assay. Each value shows mean ± SD (*n* = 3). Significance was determined by one-tailed Mann–Whitney U test; *p ≤ 0.050. *ATM* ataxia telangiectasia mutated Protein, *ATR* ataxia telangiectasia and rad3-related Protein, *DMSO* dimethyl sulfoxide, *HNSCC* head and neck squamous cell carcinoma, *NK* natural killer cells, *HPV* human papilloma virus, *IL* interleukin

To further analyze the killing process elicited by the NK cells, we examined the expression of the activation markers CD69 and CD335 on the NK cell surface. For flow cytometric measurements, the co-cultures were stained with anti-CD69 and anti-CD335 antibodies as described in the “Materials and methods” section. However, no significant changes were seen in the regulation either marker (Suppl. Fig. 1). Additionally, NK cell cytotoxicity-related molecules were analyzed in the supernatants of the co-culture experiments (Fig. 3).

Out of the 13 analyzed LEGENDplex™ Human CD8/NK Panel targets, granzyme B, IL‑6, granulysin, and INF‑γ showed significant changes. Results of the analyses of IL‑2, TNF‑α, granzyme A, and perforin are shown in Suppl. Fig. 2, but their changes were not significant, while the remaining five targets were below the detection limit. Generally, most of the significant differences were again seen in the HPV-negative HNSCC cell lines when comparing DDRi to CTRL or DDRi + RT to RT alone. In contrast, RT itself seemed to have an effect especially in Cal33 tumor cells, as downregulation of granulysin and upregulation of IL‑6 were seen when comparing the irradiated with the nonirradiated treatment group (no statistical comparison conducted). A significant downregulation was observed for granulysin through ATMi and RT + ATMi in co-culture of NK cells with Cal33 tumor cells. For the HSC4 cell line, a significant downregulation of granzyme B and granulysin by ATMi was discovered. For granulysin, only RT + ATRi and not RT + ATMi induced a significant downregulation of secretion. Further, again in the RT + ATRi treatment of HSC4, a significant downregulation of INF‑γ was observed. Interleukin‑6 was the only target which showed significant differences in both HPV-negative and HPV-positive cell lines, as it was upregulated through ATRi in a co-culture with HSC4 but downregulated through RT + ATMi in a co-culture with UM-SCC-47.

We next aimed to investigate whether the NK cell-induced tumor cell killing can be modulated by adding immune checkpoint inhibitors to the co-cultures.

### Additional immune checkpoint inhibition (durvalumab and monalizumab) did not significantly further enhance NK cell-mediated HSC4 tumor cell killing.

**Fig. 4:**
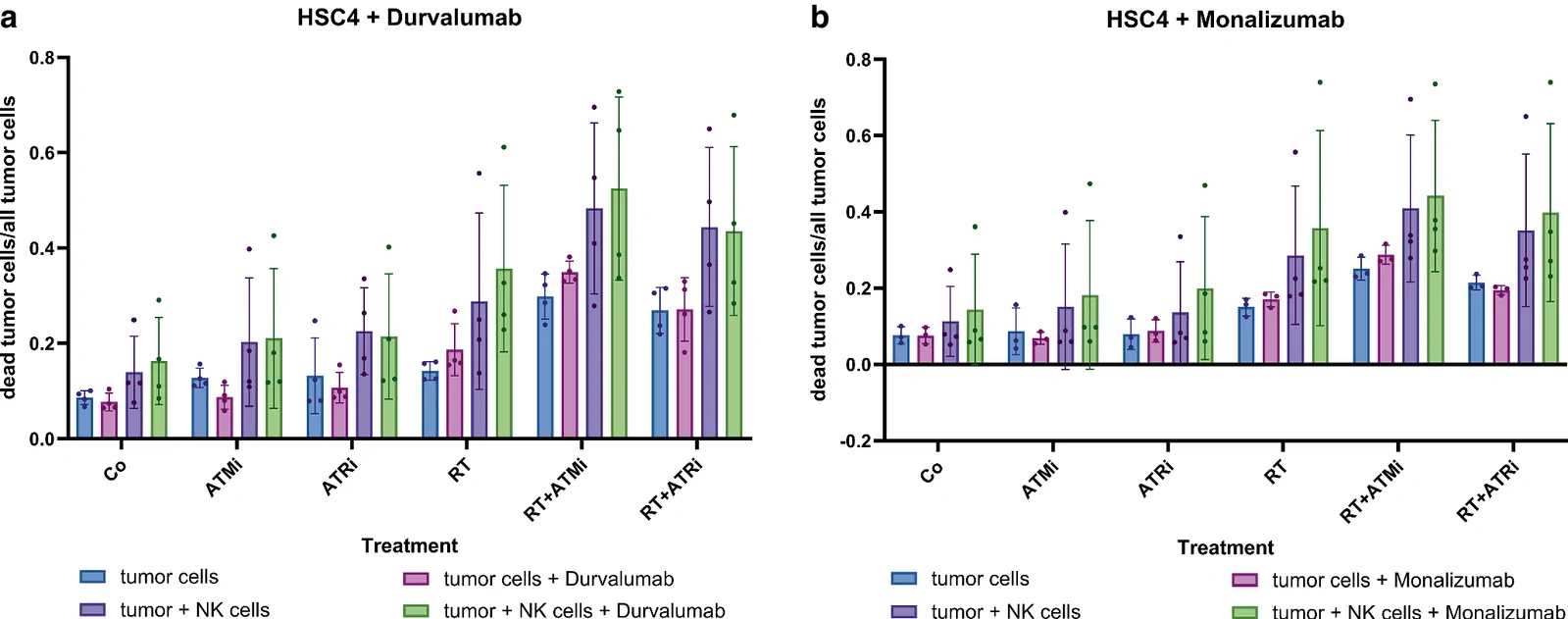
Effect of immune checkpoint inhibitors on co-cultures of pretreated HSC4 tumor cells and primary human NK cells: the HSC4 tumor cells were pretreated with either 1 µM of the ATM inhibitor AZD0156, 0.1 µM of the ATR inhibitor VE-822, RT of 2 × 5 Gy, or a combination. DMSO-exposed cell cultures served as control (*Co*). Additionally, either the PD-L1 inhibitor durvalumab (6.02 mg/mL) or the NKG2A inhibitor monalizumab (7.72 mg/mL) was added. Cell death rates were measured in each treatment group using multicolor flow cytometry, as described in Fig. 2a. **a** Cell death rates after additional treatment with durvalumab in HSC4. Each value shows mean ± SD (*n* = 4). **b** Cell death rates after additional treatment with monalizumab in HSC4. Each value shows mean ± SD (*n* = 4). *ATMi* ataxia telangiectasia mutated Protein Inhibitor, *ATRi* ataxia telangiectasia and rad3-related Protein Inhibitor, *DMSO* dimethyl sulfoxide, *HPV* human papilloma virus, *NK* natural killer cells, *RT* radiotherapy

We first tested the PD-L1 inhibitor durvalumab in co-cultures with HSC4 tumor cells (Fig. 4a), as the most significant changes in NK cell-mediated tumor cell killing were observed here (Fig. 2b). However, no increase in the NK cell killing ability was detectable in this setting. We then tested the NKG2A inhibitor monalizumab. Here again, NK cells led to an increased cell death of tumor cells but addition of monalizumab show only minimal differences (Fig. 4b). Although this NK cell-induced tumor cell killing did not reach statistical significance due to higher standard deviations in the experimental runs, pretreatment with RT + DDRi resulted in the highest overall tumor cell killing in both setups.

## Discussion

Head and neck squamous cell carcinoma, especially with a negative HPV status, is still a challenge for conventional treatment due to mutations leading to reduced radiosensitivity [7, 31]. Therefore, enhancing the sensitivity of tumor cells to radiotherapy is a current topic of research aiming to improve local treatment for patients with HPV-negative HNSCC. However, the disease should also be controlled systemically to improve patient survival. Small kinase inhibitors (smKI) targeting the DNA damage response, DDRi, are a promising option for making tumor cells on the one hand more susceptible to radiation-induced DNA damage and on the other hand for changing the immune properties of the cancer cells [8, 22]. These changes in immune phenotype not only impact parts of the acquired immune system, such as T cells, which are most studied, but also the innate immune system, including NK cells [32]. Because there is still limited knowledge of the impact that DNA-targeting therapy has on NK cells, we aimed to elucidate which role NK cells play in the early antitumor response against HNSCC tumor cells pretreated with ATMi or ATRi alone or in combination with RT.

Meidenbauer et al. showed upregulated surface expression of the immunostimulatory molecules ICM ICOS‑L and CD137‑L on HNSCC cells after combining RT with ATRi, both of which are linked with the activation and functionality of NK cells [22, 24, 25]. As NK cells are known to infiltrate tumors and trigger a direct reaction against them without the need for prior sensitization, these innate immune cells are especially interesting when looking into new therapeutic options, including immune therapies. Just recently, antibody-secreting B cells were found in HNSCC that secrete specific antitumor antibodies which are recognized by macrophages and NK cells with Fc receptors [33]. This highlights the importance of innate immune cells in anti-HNSCC tumor defense.

The results of the established ex vivo killing assay show that human NK cell-induced tumor cell killing is part of the clinically relevant early antitumor response, as we could detect it 24 h after co-culturing pretreated HNSCC with NK cells. Selection of the measurement timepoint was guided by videographic analyses of NK cell–tumor cell co-cultures conducted over a 48-hour period (not shown). These experiments demonstrated that cytotoxic activity was largely confined to the first 24 h, after which NK cell viability markedly declined, consistent with the limited stability typically observed for primary NK cells under the ex vivo model system conditions [34]. However, evaluation of the activation markers CD69 and CD335 suggests that 24 h may not constitute the most informative timepoint for assessing their regulation, as no measurable changes were detected at this stage. This discrepancy highlights the fact that while effector functions peak early, phenotypic activation markers may follow distinct kinetics that are not fully captured within the same timeframe. Marzio et al. state that CD69 is an especially early marker and can already be detected 1–2 h after stimulation [35]. However, at that timepoint, the killing activity of NK cells is limited.

With the established cytotoxicity assay, we aimed to determine whether specific combinations of hypofractionated radiotherapy and ATM or ATR inhibitors could enhance NK cell-mediated tumor cell killing. While our data align with the fact that RT combined with ATRi shows the highest toxicity in HNSCC cells regardless of HPV status [22], the NK cell-induced tumor cell killing was only significantly enhanced in one HPV-negative HNSCC cell line, i.e., the HSC4 tumor cells, but here when combining RT with ATMi. Concerning the HPV-positive cell lines, no treatment approach tested was able to significantly increase the NK cell-driven cell death rates in the co-cultures. Attempts to normalize data to donor-matched controls did not substantially reduce variability, suggesting that the observed spread predominantly reflects intrinsic donor-dependent differences in NK-cell activity. However, the results of this study suggest that under specific circumstances, treatment with ATMi and a combination of RT + ATMi is more efficacious for inducing NK cell-related tumor cell killing than treatment with ATRi or RT + ATRi. That aligns with the findings that alterations in the expression of activity-associated receptors on NK cells after co-cultivation with HNSCC tumor cells, particularly of those that were treated with RT and inhibition of ATM beforehand, are observed [28]. Since enhanced killing was observed in only one of the HPV-negative cell lines, our data suggest that HPV status alone is not the determining factor, but that additional cell line-specific characteristics are likely to influence the response. For the HSC4 cell line, a mutation in the *PTEN* gene and strong expression of *TIPE3* are known [36]. Loss of *PTEN* induces cellular senescence, consistent with our previous findings [28], and drives replication stress in proliferating epithelial cells, thereby activating a DNA damage response (DDR). Furthermore, reduced *TIPE3* expression correlates with increased malignancy and unfavorable clinical outcomes in HNSCC [37, 38]. These could be reasons for the unique treatment response of this cell line in our analyses. Therefore, a small group of carefully selected patients aligning with the criteria might benefit from treatment with ATMi rather than ATRi. Although our findings do not allow for a general recommendation to preferentially treat HPV-negative HNSCC patients with ATMi over ATRi, the potential differential benefit suggests that such treatments warrant careful consideration on an individual basis considering the huge tumor heterogeneity within HNSCC [39].

The overall trend toward enhanced NK cell-mediated tumor cell killing across all four cell lines raises the question of whether NK cell cytotoxicity could be further augmented through the blockade of distinct immune-suppressive ICM. The immune response-regulating checkpoints that tumor cells make use of to inhibit immune cells and antibodies against PD-L1 and NKG2A are discussed to overcome the NK-suppressive phenotype of tumor cells [40]. As different treatment modalities are often combined clinically, we also combined the RT + DDRi treatment with immune checkpoint inhibition in our model system. Targeting the PD-1/PD-L1 immune checkpoint axis with pembrolizumab and nivolumab has established new treatment standards regarding recurrent or metastatic HNSCC [41, 42]. We first chose to block this axis with the PD-L1 inhibitor durvalumab in co-culture with HSC4. However, we could not detect an increase in NK cell-induced tumor cell killing. We then tested the NKG2A-inhibitor monalizumab, as André et al. showed that NKG2A may represent an important checkpoint in tumor therapy [29]. But again, we did not observe significant effects in our model system, including additional experiments in the HPV-positive cell line UM-SCC-47 (Suppl. Fig. 3). Thus, NK cell-mediated tumor cell killing was largely independent of PD-1/PD-L1 or NKG2A blockade under the tested conditions.

We additionally analyzed key effector molecules associated with NK cell-mediated killing in the supernatants of the co-cultures. Natural killer cells are known to secrete granules containing granzymes, which can induce tumor cell death. They are released together with other molecules such as perforin or granulysin which help the granzymes to enter their target cells [43]. Although we anticipated that enhanced NK cell cytotoxicity would be accompanied by increased secretion of effector molecules, our data revealed that factors such as granzyme B and granulysin were downregulated in treatment groups in which cytotoxic activity was elevated. Since these effector molecules were detected in the supernatant and therefore extracellular, we took a further look into the extracellular and intracellular functions of granzyme B. Boivin et al. reported that extracellular granzyme B can occur independently of target cell release and apoptosis, for example following prolonged immune cell activation or chronic inflammatory conditions [44], which means that functions can vary according to the location where the enzymes are detected. Overall, extracellular measurement of granzyme B provides information solely on the amount secreted, whereas intracellular assessment offers additional insights into its uptake by target cells and consequent functional activity [45]. However, it must be stressed that reduced release of granzyme B and granulysin, particularly after pretreatment of HSC4 tumor cells with ATRi, was observed in comparison to ATMi, albeit without statistical significance.

While the role of granzyme B and granulysin in the cytotoxicity of NK cells is a direct one, there are other mediators, such as interleukin‑6, which have a more indirect effect on NK cell activity. Interestingly, IL‑6 was the only target regulated in a manner partly opposite to the other molecules investigated and has also been shown to reduce the cytolytic activity of NK cells before, concomitantly with the downregulation of granzyme B and perforin [46]. It can also protect cancer cells from treatment-induced DNA damage and is therefore yet another interesting target in antitumor therapy [47]. We revealed that IL‑6 was significantly upregulated through ATRi in a co-culture with HSC4. Given that NK cells are capable of inducing target cell death through multiple pathways, not all of which are dependent on granzymes, it is additionally conceivable that tumor cell killing in our setting is also mediated via alternative mechanisms [48]. Prager et al. have reported that NK cells can transition to death receptor-mediated killing over time [48].

## Conclusion

Our analyses indicate that NK cells provide a measurable contribution to the elimination of HNSCC cells following RT and DDRi pretreatment. Notably, NK cell-mediated cytotoxicity appeared to depend on cell line-specific characteristics and was more pronounced in the context of ATM inhibition. These observations suggest that a subset of patients may particularly benefit from combining RT with ATMi rather than ATRi. Future studies aimed at identifying predictive features of NK cell responsiveness will be essential to refine patient selection and maximize the therapeutic benefit.

## Supplementary Information

ESM1: Supplementary material 1

## Data Availability

All data sets and statistical analyses generated and used in this study are available upon request.

## Abbreviations

ATM: Ataxia-telangiectasia mutated
ATMi: ATM inhibitor
ATR: Ataxia telangiectasia and Rad3-related protein
ATRi: ATR inhibitor
DDR: DNA damage response
DDRi: DNA damage response inhibitor
DSB: Double-strand break
ELISA: Enzyme-linked immunosorbent assay
HNSCC: Head and neck squamous cell carcinoma
HPV: Human papillomavirus
NK: Natural killer (cells)
NKG2D: Natural killer group 2 member D
NKp30/44/46: Natural cytotoxicity receptors (NKp30, NKp44, NKp46)
PBMC: Peripheral blood mononuclear cells
PI3K: Phosphoinositide 3‑kinase
RT: Radiotherapy
SmKI(s): Small molecule kinase inhibitor(s)

## References

[1] Johnson DE, Burtness B, Leemans CR, Lui VWY, Bauman JE, Grandis JR, Head and neck squamous cell carcinoma. Nat Rev Dis Primers, 2020. 6(1): p. 92. 10.1038/s41572-020-00224-3 PMC7944998 33243986

[2] Gormley M, Creaney G, Schache A, Ingarfield K, Conway DI, Reviewing the epidemiology of head and neck cancer: definitions, trends and risk factors. Br Dent J, 2022. 233(9): p. 780–786. 10.1038/s41415-022-5166-x PMC9652141 36369568

[3] Bhatia A, Burtness B, Treating Head and Neck Cancer in the Age of Immunotherapy: A 2023 Update. Drugs, 2023. 83(3): p. 217–248. 10.1007/s40265-023-01835-2 36645621

[4] Afshari K, Sohal KS, Potential Alternative Therapeutic Modalities for Management Head and Neck Squamous Cell Carcinoma: A Review. Cancer Control, 2023. 30: p. 10732748231185003. 10.1177/10732748231185003 PMC10278411 37328298

[5] Hashibe M, Brennan P, Chuang SC et al., Interaction between tobacco and alcohol use and the risk of head and neck cancer: pooled analysis in the International Head and Neck Cancer Epidemiology Consortium. Cancer Epidemiol Biomarkers Prev, 2009. 18(2): p. 541–50. 10.1158/1055-9965.Epi-08-0347 PMC3051410 19190158

[6] Leemans CR, Snijders PJF, Brakenhoff RH, The molecular landscape of head and neck cancer. Nat Rev Cancer, 2018. 18(5): p. 269–282. 10.1038/nrc.2018.11 29497144

[7] Zhou C, Parsons JL, The radiobiology of HPV-positive and HPV-negative head and neck squamous cell carcinoma. Expert Rev Mol Med, 2020. 22: p. e3. 10.1017/erm.2020.4 PMC7754878 32611474

[8] Glorieux M, Dok R, Nuyts S, Novel DNA targeted therapies for head and neck cancers: clinical potential and biomarkers. Oncotarget, 2017. 8(46): p. 81662–81678. 10.18632/oncotarget.20953 PMC5655317 29113422

[9] Faulhaber EM, Jost T, Symank J, Scheper J, Bürkel F, Fietkau R, Hecht M, Distel LV, Kinase Inhibitors of DNA-PK, ATM and ATR in Combination with Ionizing Radiation Can Increase Tumor Cell Death in HNSCC Cells While Sparing Normal Tissue Cells. Genes (Basel), 2021. 12(6). 10.3390/genes12060925 PMC8235750 34204447

[10] Priya B, Ravi S, Kirubakaran S, Targeting ATM and ATR for cancer therapeutics: Inhibitors in clinic. Drug Discov Today, 2023. 28(8): p. 103662. 10.1016/j.drudis.2023.103662 37302542

[11] Chan Wah Hak CML, Rullan A, Patin EC, Pedersen M, Melcher AA, Harrington KJ, Enhancing anti-tumour innate immunity by targeting the DNA damage response and pattern recognition receptors in combination with radiotherapy. Front Oncol, 2022. 12: p. 971959. 10.3389/fonc.2022.971959 PMC9465159 36106115

[12] AstraZeneca. Study to Assess the Safety and Preliminary Efficacy of AZD0156 at Increasing Doses Alone or in Combination With Other Anti-cancer Treatment in Patients With Advanced Cancer (AToM). 2022 19. September 2025]; Available from: https://clinicaltrials.gov/study/NCT02588105.

[13] Scheper J, Hildebrand LS, Faulhaber EM et al., Tumor-specific radiosensitizing effect of the ATM inhibitor AZD0156 in melanoma cells with low toxicity to healthy fibroblasts. Strahlenther Onkol, 2023. 199(12): p. 1128–1139. 10.1007/s00066-022-02009-x PMC10673781 36229655

[14] Riches LC, Trinidad AG, Hughes G et al., Pharmacology of the ATM Inhibitor AZD0156: Potentiation of Irradiation and Olaparib Responses Preclinically. Mol Cancer Ther, 2020. 19(1): p. 13–25. 10.1158/1535-7163.Mct-18-1394 31534013

[15] KGaA MH. Berzosertib Human Mass Balance Study (DDRiver Solid Tumors 208). 2023 19. September 2025]; Available from: https://clinicaltrials.gov/study/NCT05246111.

[16] EMD Serono Research & Development Institute I. Berzosertib + Topotecan in Relapsed Platinum-Resistant Small-Cell Lung Cancer (DDRiver SCLC 250). 2023 19. September 2025]; Available from: https://clinicaltrials.gov/study/NCT04768296.

[17] Taniguchi H, Chakraborty S, Takahashi N et al., ATR inhibition activates cancer cell cGAS/STING-interferon signaling and promotes antitumor immunity in small-cell lung cancer. Sci Adv, 2024. 10(39): p. eado4618. 10.1126/sciadv.ado4618 PMC11430494 39331709

[18] Bruciamacchie M, Garambois V, Vie N et al., ATR inhibition potentiates FOLFIRINOX cytotoxic effect in models of pancreatic ductal adenocarcinoma by remodelling the tumour microenvironment. Br J Cancer, 2025. 132(2): p. 222–235. 10.1038/s41416-024-02904-3 PMC11746931 39613844

[19] Cote GM, Kochupurakkal BS, Do K et al., A Translational Study of the ATR Inhibitor Berzosertib as Monotherapy in Four Molecularly Defined Cohorts of Advanced Solid Tumors. Clin Cancer Res, 2025. 31(1): p. 35–44. 10.1158/1078-0432.Ccr-24-1867 39453756

[20] Dillon MT, Patin EC, Mohammed K et al., Tumor control and immune activation through palliative irradiation and ATR inhibition, PATRIOT Part C: a phase Ib trial. Nat Commun, 2025. 16(1): p. 7064. 10.1038/s41467-025-62249-0 PMC12316918 40750587

[21] Dobler C, Jost T, Hecht M, Fietkau R, Distel L, Senescence Induction by Combined Ionizing Radiation and DNA Damage Response Inhibitors in Head and Neck Squamous Cell Carcinoma Cells. Cells, 2020. 9(9). 10.3390/cells9092012 PMC7563880 32883016

[22] Meidenbauer J, Wachter M, Schulz SR, Mostafa N, Zülch L, Frey B, Fietkau R, Gaipl US, Jost T, Inhibition of ATM or ATR in combination with hypo-fractionated radiotherapy leads to a different immunophenotype on transcript and protein level in HNSCC. Front Oncol, 2024. 14: p. 1460150. 10.3389/fonc.2024.1460150 PMC11473424 39411143

[23] Sun R, Lee KY, Mei Y, Nickles E, Le Lin J, Xia R, Liu H, Schwarz H, Induction of cell death in malignant cells and regulatory T cells in the tumor microenvironment by targeting CD137. Oncoimmunology, 2025. 14(1): p. 2443265. 10.1080/2162402x.2024.2443265 PMC12952740 39716931

[24] Montes-Casado M, Ojeda G, Aragoneses-Fenoll L, López D, de Andrés B, Gaspar ML, Dianzani U, Rojo JM, Portolés P, ICOS deficiency hampers the homeostasis, development and function of NK cells. PLoS One, 2019. 14(7): p. e0219449. 10.1371/journal.pone.0219449 PMC6613708 31283790

[25] Matson AW, Hullsiek R, Dixon KJ et al., Enhanced IL-15-mediated NK cell activation and proliferation by an ADAM17 function-blocking antibody involves CD16A, CD137, and accessory cells. J Immunother Cancer, 2024. 12(7). 10.1136/jitc-2024-008959 PMC11284835 39053944

[26] Sivori S, Vacca P, Del Zotto G, Munari E, Mingari MC, Moretta L, Human NK cells: surface receptors, inhibitory checkpoints, and translational applications. Cell Mol Immunol, 2019. 16(5): p. 430–441. 10.1038/s41423-019-0206-4 PMC6474200 30778167

[27] Cózar B, Greppi M, Carpentier S, Narni-Mancinelli E, Chiossone L, Vivier E, Tumor-Infiltrating Natural Killer Cells. Cancer Discov, 2021. 11(1): p. 34–44. 10.1158/2159-8290.Cd-20-0655 PMC7611243 33277307

[28] Jost T, Wachter M, Meidenbauer J, Fietkau R, Gaipl US, Inhibiting the DNA damage repair of HNSCC cells in combination with normo-fractionated radiotherapy influences clonogenicity, senescence and expression of NK cell activation markers. Sci Rep, 2025. 15(1): p. 31827. 10.1038/s41598-025-17858-6 PMC12397261 40883442

[29] André P, Denis C, Soulas C et al., Anti-NKG2A mAb Is a Checkpoint Inhibitor that Promotes Anti-tumor Immunity by Unleashing Both T and NK Cells. Cell, 2018. 175(7): p. 1731–1743.e13. 10.1016/j.cell.2018.10.014 PMC6292840 30503213

[30] Patin EC, Nenclares P, Chan Wah Hak C et al., Sculpting the tumour microenvironment by combining radiotherapy and ATR inhibition for curative-intent adjuvant immunotherapy. Nat Commun, 2024. 15(1): p. 6923. 10.1038/s41467-024-51236-6 PMC11319479 39134540

[31] Rieckmann T, Tribius S, Grob TJ, Meyer F, Busch CJ, Petersen C, Dikomey E, Kriegs M, HNSCC cell lines positive for HPV and p16 possess higher cellular radiosensitivity due to an impaired DSB repair capacity. Radiother Oncol, 2013. 107(2): p. 242–6. 10.1016/j.radonc.2013.03.013 23602369

[32] Li J, Yang H, Zhu M, Zhang P, Liu Y, Niu Y, Zhou T, Li Y, Unlocking the therapeutic potential of the STING signaling pathway in anti-tumor treatment. Clin Exp Med, 2025. 25(1): p. 290. 10.1007/s10238-025-01838-1 PMC12343656 40794212

[33] Sameshima J, Chen H, Kaneko N et al., Tumor-infiltrating B cells produce tumor-specific antibodies and may contribute to suppressing tumor in head and neck squamous cell carcinoma. Oncoimmunology, 2025. 14(1): p. 2543019. 10.1080/2162402x.2025.2543019 PMC12952742 40778950

[34] Martin-Iglesias S, Herrera L, Santos S, Vesga M, Eguizabal C, Lanceros-Mendez S, Silvan U, Analysis of the impact of handling and culture on the expansion and functionality of NK cells. Front Immunol, 2023. 14: p. 1225549. 10.3389/fimmu.2023.1225549 PMC10451065 37638054

[35] Marzio R, Mauël J, Betz-Corradin S, CD69 and regulation of the immune function. Immunopharmacol Immunotoxicol, 1999. 21(3): p. 565–82. 10.3109/08923979909007126 10466080

[36] Li H, Wawrose JS, Gooding WE, Garraway LA, Lui VW, Peyser ND, Grandis JR, Genomic analysis of head and neck squamous cell carcinoma cell lines and human tumors: a rational approach to preclinical model selection. Mol Cancer Res, 2014. 12(4): p. 571–82. 10.1158/1541-7786.Mcr-13-0396 PMC3989421 24425785

[37] Parisotto M, Grelet E, El Bizri R, Dai Y, Terzic J, Eckert D, Gargowitsch L, Bornert JM, Metzger D, PTEN deletion in luminal cells of mature prostate induces replication stress and senescence in vivo. J Exp Med, 2018. 215(6): p. 1749–1763. 10.1084/jem.20171207 PMC5987915 29743291

[38] Chen W, Chen X, Wang L et al., TIPE3 represses head and neck squamous cell carcinoma progression via triggering PGAM5 mediated mitochondria dysfunction. Cell Death Dis, 2023. 14(4): p. 251. 10.1038/s41419-023-05775-3 PMC10079926 37024453

[39] Zhou Y, Wang L, Liu M, Jiang H, Wu Y, Oral squamous cell carcinoma: Insights into cellular heterogeneity, drug resistance, and evolutionary trajectories. Cell Biol Toxicol, 2025. 41(1): p. 101. 10.1007/s10565-025-10048-0 PMC12162747 40504271

[40] Cerwenka A, Kopitz J, Schirmacher P, Roth W, Gdynia G, HMGB1: The metabolic weapon in the arsenal of NK cells. Mol Cell Oncol, 2016. 3(4): p. e1175538. 10.1080/23723556.2016.1175538 PMC4972106 27652323

[41] Ferris RL, Blumenschein G, Jr., Fayette J et al., Nivolumab for Recurrent Squamous-Cell Carcinoma of the Head and Neck. N Engl J Med, 2016. 375(19): p. 1856–1867. 10.1056/NEJMoa1602252 PMC5564292 27718784

[42] Harrington KJ, Burtness B, Greil R et al., Pembrolizumab With or Without Chemotherapy in Recurrent or Metastatic Head and Neck Squamous Cell Carcinoma: Updated Results of the Phase III KEYNOTE-048 Study. J Clin Oncol, 2023. 41(4): p. 790–802. 10.1200/jco.21.02508 PMC9902012 36219809

[43] Krzewski K, Coligan JE, Human NK cell lytic granules and regulation of their exocytosis. Front Immunol, 2012. 3: p. 335. 10.3389/fimmu.2012.00335 PMC3494098 23162553

[44] Boivin WA, Cooper DM, Hiebert PR, Granville DJ, Intracellular versus extracellular granzyme B in immunity and disease: challenging the dogma. Lab Invest, 2009. 89(11): p. 1195–220. 10.1038/labinvest.2009.91 PMC7102238 19770840

[45] Hagn M, Sutton VR, Trapani JA, A colorimetric assay that specifically measures Granzyme B proteolytic activity: hydrolysis of Boc-Ala-Ala-Asp-S-Bzl. J Vis Exp, 2014(93): p. e52419. 10.3791/52419 PMC4354391 25489668

[46] Kang YJ, Jeung IC, Park A, Park YJ, Jung H, Kim TD, Lee HG, Choi I, Yoon SR, An increased level of IL‑6 suppresses NK cell activity in peritoneal fluid of patients with endometriosis via regulation of SHP‑2 expression. Hum Reprod, 2014. 29(10): p. 2176–89. 10.1093/humrep/deu172 25035432

[47] Kumari N, Dwarakanath BS, Das A, Bhatt AN, Role of interleukin‑6 in cancer progression and therapeutic resistance. Tumour Biol, 2016. 37(9): p. 11553–11572. 10.1007/s13277-016-5098-7 27260630

[48] Prager I, Liesche C, van Ooijen H et al., NK cells switch from granzyme B to death receptor-mediated cytotoxicity during serial killing. J Exp Med, 2019. 216(9): p. 2113–2127. 10.1084/jem.20181454 PMC6719417 31270246

