# Supplementary material for: Radiotherapy and DNA damage response inhibitors modestly sensitize HNSCC to NK cell killing, with ATM inhibition more effective than ATR inhibition
Source: Strahlenther Onkol. 2026 Apr 17;202(10):1071–82. doi: 10.1007/s00066-026-02522-3 (PMC13597543; doi:10.1007/s00066-026-02522-3)
Supplement: Supplementary file 1 [file 66_2026_2522_MOESM1_ESM.docx]

**Supplementary Figure S1:** **Analysis of NK cell activation markers after co-cultivation with pre-treated HNSCC for 24 hours.** The HNSCC cell lines Cal33, HSC4, UM-SCC-47 and UD-SCC-2 were pretreated with either 1 µM of the ATM-inhibitor AZD0156, 0.1 µM of the ATR-inhibitor VE-822, RT of 2 x 5 Gy or a combination of smKI and RT and DMSO as a vehicle control.

**Expression of CD69 and CD335 on the NK cell surface:** expression was measured using multicolor flow-cytometry. Each value shows mean ± SD (Cal33, *n* = 4; HSC4, *n* = 5; UM-SCC-47, *n* = 4; UD-SCC-2, *n* = 4).

**Supplementary material**

**Supplementary Figure S2: Secretion of NK cell related molecules after co-cultivation with pre-treated HNSCC for 24 hours.** The HNSCC cell lines Cal33, HSC4, UM-SCC-47 and UD-SCC-2 were pretreated with either 1 µM of the ATM-inhibitor AZD0156, 0.1 µM of the ATR-inhibitor VE-822, RT of 2 x 5 Gy or a combination of smKI and RT and DMSO as a vehicle control. Secretion Granzyme A, IL-2, Perforin and TNF-α was analysed using a bead-based Multiplex Assay. Each value shows mean ± SD (*n* = 3).

**Supplementary Figure S3: Effect of the immune-checkpoint-inhibitor Monalizumab on the co-culture of pre-treated UM-SCC-47 tumor cells and primary human NK cells:** the UM-SCC-47 tumor cells were pretreated with either 1 µM of the ATM-inhibitor AZD0156, 0.1 µM of the ATR-inhibitor VE-822, RT of 2 x 5 Gy or a combination. DMSO exposed cell cultures served as control (Co). Additionally, NKG2A-inhibitor Monalizumab (conc. 7.72 mg / mL) was added. Cell death rates were measured in each treatment group by using multicolor flow-cytometry as described in **Figure 2A.**
